# Trends and correlates of maternal, newborn and child health services utilization in primary healthcare facilities: an explorative ecological study using DHIMSII data from one district in the Volta region of Ghana

**DOI:** 10.1186/s12884-020-03195-1

**Published:** 2020-09-17

**Authors:** Robert Kaba Alhassan, Seth Owusu-Agyei, Evelyn Korkor Ansah, Margaret Gyapong, Anthony Ashinyo, Mary Eyram Ashinyo, Edward Nketiah-Amponsah, Edem Akorli-Adzimah, Edith Ekpor

**Affiliations:** 1 Institute of Health Research, University of Health and Allied Sciences, Ho, Ghana; 2 Ghana AIDS/STI Control Programme, Ghana Health Service, Accra, Ghana; 3 Department of Quality Assurance and Safety, Ghana Health Service, Accra, Ghana; 4 Department of Economics, University of Ghana, Legon Accra, Ghana; 5 Ho West District Health Directorate, Volta Region Ho, Ghana

**Keywords:** Continuum of care, District health information management system II, Facility-based spontaneous vaginal deliveries, Maternal, Newborn and child health, Child immunizations, Utilization, Primary healthcare, Antenatal care, Health policy, Ghana

## Abstract

**Background:**

Sustainable Development Goal 3 aims at reducing global neonatal mortality to at least 12 per 1000 livebirths, under-five mortality to at least 25 per 1000 livebirths and maternal mortality ratio to less than 70 per 100,000 livebirths by 2030. Considering the achievement so far, many countries in sub-Saharan Africa, including Ghana are not likely to achieve these targets. Low utilization of maternal, newborn and child health (MNCH) services partly account for this predicament. This study explored the trend and correlates of MNCH services utilization in one administrative district in the Volta Region of Ghana.

**Methods:**

This is an explorative ecological study employing trend analysis of 2015–2017 data from Ghana Health Service District Health Information Management System II. Univariate Poisson regression models were used to determine the factors associated with MNCH services utilization at 95% confidence level.

**Results:**

Cumulative record of 17,052 antenatal care (ANC) attendance and 2162 facility-based spontaneous vaginal deliveries (SVDs) was discovered. Compelling evidence of potential unskilled deliveries was observed in 23% of the 26 facilities reported in the DHIMSII data. High cumulative number of midwives in health facilities associated positively with high records of ANC visits (IRR = 1.30, [95% CI:1.29, 1.32]; *p* = 0.0001), facility-based SVDs (IRR = 1.30 [95% CI:1.25, 1.35]; *p* = 0.0001) and BCG immunizations (IRR = 1.32 [95% CI:1.29, 1.34]; *p* = 0.0001). Likewise, high records of ANC visits correlated positively with high facility-based SVDs and child immunizations records (*p* < 0.0001).

**Conclusion:**

Targeted health system and community level interventions alongside progressive frontline health staff motivation and retention strategies could further enhance enrollment and retention of mothers in pre-natal and postnatal care services throughout the continuum of care to guarantee better MNCH health outcomes. Investments in universal coverage for quality ANC services has the potential to enhance utilization of supervised deliveries and post-natal care services such as immunizations.

## Background

Maternal, newborn and child health (MNCH) are important indicators of health systems’ performance across the globe [1]. Sustainable Development Goal (SDG) 3 aims at reducing global neonatal mortality to at least 12 per 1000 livebirths, under-five mortality to at least 25 per 1000 livebirths and maternal mortality ratio to less than 70 per 100,000 livebirths by 2030 [1]. Unfortunately, many countries in sub-Saharan Africa, including Ghana, are not likely to achieve these targets before the end of 2030 [2, 3].

In 2016, maternal mortality ratio (MMR) in the WHO African Region was estimated to be 542 per 100,000 livebirths compared to 16 per 100,000 in Europe. MMR in sub-Saharan Africa is projected to improve marginally to 347 per 100,000 by 2030 which is 34 times higher than the average MMR ratio in Europe [2]. The current pace of decline in maternal mortality in Africa suggests that the continent will not attain the MMR target of 70 per 100,000 livebirths until 2084 [2].

Additionally, the proportion of facility-based deliveries and births attended to by skilled health personnel in Africa remains lowest in the world, recording an average rate of 59% between 2013 and 2018 compared to 99% in Europe, 97% in Western Pacific, 95% in the Americas, 81% in South-East Asia and 79% in Eastern Mediterranean region [2].

Ghana continues to experience unacceptable levels of maternal, neonatal and child mortalities coupled with low immunization coverage for vaccine preventable childhood diseases. Per Ghana’s current neonatal mortality of 29 per 1000 livebirths, it is not likely the 2030 target of 12 per 1000 live births will be met [4]. Similarly, the WHO target of 70 per 100,000 by the year 2030 will be missed if there are no improvements in maternal, newborn and child health (MNCH) services [2]. As at 2016, the proportion of pregnant women who had skilled supervised deliveries was 71%, far below the SDG target of 90%, even though better than the African average of 54% [2].

Immunization coverage for Diphtheria Pertussis Tetanus (DPT) or Pentavalent3 (Penta3) in Ghana in 2016 was 93% with Penta1 coverage of 94% [2]. The proportion of children aged < 1 year who received up to three doses of polio3 vaccine was 95% in 2016 above the African average of 73%; likewise, coverage of Bacillus Calmette Guerin (BCG) and measles vaccine were 94 and 89% respectively compared to the WHO African region averages of 81 and 72% respectively [2].

Notwithstanding the progressive efforts towards increasing accessibility and utilization of MNCH services in Ghana, there are rural-urban gaps in access to basic healthcare services throughout the continuum of care (CoC) for MNCH services. Volta Region is one of the relatively under-resourced regions in Ghana in terms of health infrastructure with undesirable health outcome indicators. As per the Expanded Programme on Immunization (EPI) schedule in Ghana (see Table 1), the Volta region is among the worst performing regions in the country. In 2018, the region recorded the lowest measles immunization coverage of 80.7%, below the national average of 92.4% [4].

**Table 1:** Expanded Programme on Immunization (EPI) schedules in Ghana

Num.	Vaccine	Age	Dose	Route/site of administration	Disease(s)
1	BCG	At birth	0.05 ml	Intradermal right upper arm	Tetanus
2	OPV0	At birth	2drops	Oral	Poliomyelitis
1	DPT-HepB-Hib1	6 weeks	0.5 ml	IM, lateral part of left thigh	Diphtheria, pertussis, Tetanus, Hepatitis B and Haemophilus Influenza type B
2	OPV1	6 weeks	2drops	Oral	Poliomyelitis
3	Pneumo 1	6 weeks	0.5 ml	IM, lateral part of right thigh	Pneumococcal diseases (pneumonia)
4	Rota 1	6 weeks	1.5 ml vial	Oral	Diarrhoea
1	OPV2	10 weeks	2drops	Oral	Poliomyelitis
2	DPT-HepB-Hib2	10 weeks	0.5 ml	IM, lateral part of left thigh	Diphtheria, pertussis, Tetanus, Hepatitis B and Haemophilus Influenza type B
3	Pneumo 2	10 weeks	0.5 ml	IM, lateral part of right thigh	Pneumococcal diseases (pneumonia)
4	Rota 2	10 weeks	1.5 ml vial	Oral	Diarrhoea
1	OPV3	14 weeks	2drops	Oral	Poliomyelitis
2	DPT-HepB-Hib3	14 weeks	0.5 ml	IM, lateral part of left thigh	Diphtheria, pertussis, Tetanus, Hepatitis B and Haemophilus Influenza type B
3	Pneumo 3	14 weeks	0.5 ml	IM, lateral part of right thigh	Pneumococcal diseases (pneumonia)
4	Rota 3	14 weeks	1.5 ml vial	Oral	Diarrhoea
1	Vitamin A	6 months	100,000 IU	Oral	Diet supplementation
2	Measles-Rubella	9 months	0.5 ml	Subcutaneous, left upper arm	Measles, Rubella
3	Yellow fever	9 months	0.5 ml	Subcutaneous, right upper arm	Yellow fever
1	Vitamin A	12 months	200,000 IU	Oral	Diet supplementation
1	Measles	18 months	0.5 ml	Subcutaneous, left upper arm	Measles
2	Vitamin A	18 months	200,000 IU	Oral	Diet supplementation

**Source:** Ghana Health Service/Ministry of Health Expanded Programme on Immunization (2020)

**Note:** After 18 months, Vitamin A is given every 6 months until the child is 5 years old

At 18 months, *LLINs* Long lasting Insecticide Treated Nets is given to the child

Similarly, Penta3 (DPT3) immunization coverage in the Volta Region in 2018 was 86.3%, below the national average of 97.3%. The region performed below the national average in Oral Polio Vaccine 3 (OPV3) and BCG immunization coverage [4] and recorded the lowest ANC coverage of 68.8% in 2016 while the percentage of supervised deliveries was 43.7% compared to the national average of 56.2% [4].

In terms of health sector human resources, the doctor to patient ratio in the Volta Region in 2018 was 1: 11, 857 compared to the national average of 1: 7058. Nurse: patient ratio was estimated to be 1: 567, relative to the national average of 1: 508 [4] and midwife to WIFA population ratio was 1: 728 compared to the national average of 1: 677 [4].

This paper explored the trends and correlates of MNCH services utilization throughout the continuum of care (CoC) in 26 primary healthcare facilities in Ho West District (HWD) of the Volta Region of Ghana.

## Methods

### Study design

The study is an explorative ecological design using secondary data from the Ghana Health Service (GHS) District Health Information Management System II (DHIMSII), 2015–2017. The DHIMSII data comprises of 26 primary healthcare facilities (i.e. clinics, health centres and functional community-based health planning and services (CHPS)) located in urban and rural communities in the HDW. These facilities comprised of both private and public facilities.

Functional CHPS compounds are the basic unit of Ghana’s health service delivery system where there is usually a trained resident Community Health Officer (CHO) (i.e. community health nurse, midwife or general nurse). Functional CHPS compounds turn to render more health services including MNCH services than the CHPS zones which are often coterminous with electoral areas within districts. In this paper, the use of CHPS compounds mainly connotes functional CHPS compounds excluding CHPS zones.

### Study setting, population and data sources

Data used for analyses was mainly from HWD, one of the 25 administrative districts in the Volta Region. Volta Region has a population of 2,118,252 (48% males and 52% females), representing approximately 7% of the estimated 30,380,482 million people in Ghana (cited in GHS, [4]). The region has 732 healthcare facilities comprising of one (1) teaching hospital, 30 hospitals, 45 clinics, 156 health centres, 14 maternity homes, 4 polyclinics and 482 CHPS compounds. HWD has an estimated population of 94,600, representing 5% the regional population [4].

The DHIMSII ecological data comprised mainly of primary healthcare facilities that render spontaneous vaginal deliveries (SVDs), antenatal care (ANC), postnatal care (PNC) and child immunizations services. The Ghana Health Service (GHS) pyramidal levels of care classify clinics, polyclinics, health centres and functional CHPS compounds as primary healthcare facilities with a mandate to manage minor and uncomplicated medical conditions. Conditions from this level of care are referred to district and regional hospitals, which in turn refer complicated cases to tertiary facilities such as the teaching hospitals. At the time of conducting this study, the total number of primary health care facilities in the HWD were 27, but 26 of these had complete information in DHIMSII from 2015 to 2017 (see Supplementary File_1).

### Sampling procedure

Employing the census technique, data from all eligible primary healthcare facilities (*N* = 26) in the HWD district was retrieved from DHIMSII, cleaned and analyzed. Data from the DHIMSII is reported cumulatively from all facilities within the district. In view of this, the unit of analysis was the health facilities with pooled data (not individual patient level) to avoid committing an ecological fallacy. The DHIMSII data was analyzed using cumulative number of SVDs, ANC attendance and immunizations for BCG, OPV1, OPV3, Penta-1, Penta-3, Yellow Fever and Measles from 2015 to 2017. Background information on facility ownership, level of care, location, catchment population of women in fertile age (WIFA), number of midwives and other cadre of frontline health staff was extracted and analyzed. Only health facilities under the jurisdiction/supervision of Ho West District Health Management Team (DHMT) were considered eligible for inclusion into this study.

### Data extraction and analysis

Data on MNCH service indicators was retrieved from DHIMS II with the assistance of the district health information officer using an extraction form developed by the researchers. Data validation was carried out by the District Director of Health of HWD at the time of the study. Extracted DHIMSII data was cleaned and coded in Microsoft Excel and later exported to STATA statistical analysis software (version 12.0) for analysis. Facility names were anonymized with codes to maintain privacy and confidentiality. Summary statistics were generated and test for differences between groups was done using the independent t-test. Univariate Poisson regression models were employed to predict determinants of utilization of MNCH services.

Missing data was handled mainly through deletion since the data set was an ecological/cluster secondary data that did not make room for imputation as part of the missing data management process. Thus, prior to the final analysis, listwise deletion was done for missing observations (i.e. one out of the 27 health facilities without DHIMSII required data). Additionally, column (variable) deletion was done for missing variables.

### Outcome variables for Poisson regression models

Poisson regression was employed to predict the main outcome variables of interest since the latter are all count variables with non-negative values. Likewise, the outcome variables are not over-dispersed and do not have excessive number of zeros [5]. The outcome variables of interest were the pooled facility-based number of SVDs, number of ANC visits and child immunizations (BCGs, OPV1, OPV3, Penta-1, Penta-3, Yellow Fever and Measles immunization). Some of the immunization schedules were not recorded in the DHIMSII hence were not included in the analysis.

Incidence Rate Ratio (IRR) was used as the output reporting option in place of the default coefficients. IRRs are transformed estimated coefficients, standard errors and confidence intervals [5] and since they only affect how results are displayed and not how they are estimated or stored, they were deemed appropriate. The exposure option was not accounted for in the Poisson regression models, without risking estimation bias, because all observations in the pooled dataset had the same and consistent data reporting from 2015 to 2017.

Even though count variables are sometimes log-transformed and analyzed using Ordinary Least Squares (OLS) regression, this statistical test was not considered on this occassion because of its peculiar limitations in count data estimations [5]. Use of OLS in count data could result in loss of data due to generation of undefined values which has the propensity of producing biased estimates [6].

### Covariates of the Poisson regression models

Independent variables loaded in the Poisson regression models were: facility ownership, location, number of midwives per health facility, number of other health staff per health facility, WIFA population and number of ANC visits in a health facility per year. Multicollinearity diagnostics were conducted on all explanatory variables before fitting them into the regression models and those with variance inflation factors (VIFs) more than the 10.0 rule of thumb [6] were dropped. “Facility ownership” was therefore dropped for recording VIF above 10.0. Statistical significance was set at 95% for all analysis.

## Results

### Situational analysis of health facilities

Out of the population of 27 eligible health facilities, 26 of the them were included, because they had the required complete DHIMSII data, resulting in data completeness rate of 96%. Situational analysis of the health facilities showed that 2 out of the 26 facilities (8%) were urban with the remaining (92%) being rural. Likewise, 2 out of the 26 facilities (2%) were privately owned with the bulk 24 (92%) being publicly owned. In terms of the level of care, Tables 2 & 3 show that health centres dominated with 46% (12/26) followed by CHPS compounds 39% (10/26) and then clinics 15% (4/26). Cumulative number of BCGs recorded in all the 26 facilities from 2015 to 2017 was 10,787 as per the DHIMSII data; records of subsequent immunizations given from 6 weeks in the continuum of care (CoC) were comparatively higher, as shown in Table 3.

**Table 2:** Absolute cumulative maternal health service utilization per facility and corresponding staff population (2015–2017)

SN	Location	Ownership	Level	SVDs (N)	ANC Attendance (N)	SVDs per midwife^a^	WIFA population (N)	Midwives per clinic (N)	Other staff per clinic (N)
1	Rural	Public	Clinic	0	0	0	0	0	3
2	Rural	Private	Clinic	221	513	74	0	3	6
3	Rural	Public	CHPS Compound	7	199	0	704	0	3
4	Rural	Public	Health Centre	42	369	21	1161	2	9
5	Rural	Public	Health Centre	148	1240	49	1175	3	12
6	Rural	Public	Health Centre	191	1350	64	1227	3	12
7	Rural	Public	Health Centre	13	160	7	1428	2	6
8	Rural	Public	CHPS Compound	0	93	0	1839	0	6
9	Rural	Public	Health Centre	38	389	19	2134	2	6
10	Rural	Public	Health Centre	7	145	0	2323	0	6
11	Rural	Public	CHPS Compound	20	304	10	2706	2	6
12	Rural	Public	CHPS Compound	271	617	136	2828	2	6
13	Rural	Public	CHPS Compound	47	606	0	2955	0	6
14	Rural	Public	CHPS Compound	30	339	0	3087	0	6
15	Rural	Public	Health Centre	219	1550	110	3108	2	21
16	Rural	Public	Health Centre	83	353	42	3293	2	15
17	Rural	Public	CHPS Compound	13	179	0	3471	0	6
18	Rural	Public	CHPS Compound	0	290	0	3604	0	6
19	Rural	Public	Health Centre	34	451	34	3654	1	9
20	Rural	Public	Health Centre	108	749	36	3848	3	21
21	Rural	Public	Health Centre	39	474	20	3989	2	24
22	Rural	Public	CHPS Compound	3	237	0	4729	0	6
23	Rural	Public	CHPS Compound	0	525	0	5411	0	6
24	Urban	Public	Clinic	445	3539	37	6302	12	0
25	Urban	Public	Health Centre	183	2151	46	13,931	4	38
26	Rural	Private	Clinic	0	230	0	78,906	3	21
**Total**				**2162**	**17,052**	**45**	**157,811**	**48**	13

Source: GHS/DHIMS II (Ho West District, 2015–2017)

Legend: *ANC* (Antenatal Care); *SVDs* (Spontaneous Vaginal Deliveries); *WIFA* (Women in Fertility Age); *CHPS* (Community-based Health Planning and Services); N (pooled absolute number recorded from 2015 to 2017); ^a^SVDs per midwife estimated by dividing absolute number of SVDs per health facility from 2015 to 2017 over the absolute number of midwives in the pertinent health facility over the same time period (2015–2017)

**Table 3:** Absolute cumulative child immunizations per health facility (2015–2017)

SN.	Level	Location	Ownership	BCG	OPV1	OPV3	Penta 1	Penta 3	Yellow Fever	Measles
1	Clinic	Rural	Public	0	0	0	0	0	0	0
2	Clinic	Rural	Private	0	0	0	0	0	0	0
3	CHPS Compound	Rural	Public	0	0	0	0	0	0	0
4	Clinic	Rural	Private	0	0	0	0	0	0	0
5	CHPS Compound	Rural	Public	84	126	151	126	126	135	150
6	Health Centre	Rural	Public	111	119	129	119	119	138	143
7	Health Centre	Rural	Public	157	234	183	234	234	206	200
8	Health Centre	Rural	Public	183	236	272	236	236	214	209
9	Health Centre	Rural	Public	233	241	339	247	247	252	247
10	Health Centre	Rural	Public	293	323	311	325	325	266	261
11	Health Centre	Rural	Public	324	367	396	377	377	349	376
12	CHPS Compound	Rural	Public	338	433	485	448	448	415	419
13	CHPS Compound	Rural	Public	365	391	369	392	392	419	409
14	CHPS Compound	Rural	Public	472	434	549	445	445	490	481
15	CHPS Compound	Rural	Public	504	563	601	563	563	553	539
16	Health Centre	Rural	Public	526	559	574	557	557	543	527
17	Health Centre	Rural	Public	536	523	531	536	536	526	515
18	Health Centre	Rural	Public	544	519	588	527	527	552	523
19	CHPS Compound	Rural	Public	562	519	576	519	519	482	453
20	CHPS Compound	Rural	Public	593	619	594	626	626	572	541
21	Health Centre	Rural	Public	599	625	813	655	655	623	615
22	Health Centre	Rural	Public	619	648	614	636	636	656	675
23	CHPS Compound	Rural	Public	874	715	714	734	734	674	720
24	CHPS Compound	Rural	Public	893	706	646	751	751	784	785
25	Health Centre	Urban	Public	964	1298	1280	1301	1301	1487	1374
26	Clinic	Urban	Public	1013	1057	1093	1057	1057	1089	1089
**Total**				**10,787**	**11,255**	**11,808**	**11,411**	**11,411**	**11,425**	**11,251**

Source: GHS/DHIMS II (Ho West District, 2015–2017)

Legend: *ANC* (Antenatal Care); *SVDs* (Spontaneous Vaginal Deliveries); *WIFA* (Women in Fertility Age); *OPV1* (Oral Polio Vaccine 1); *OPV3* (Oral Polio Vaccine 3); *Penta1* (Pentavalent 1); *Penta 3* (Pentavalent 3); *CHPS* (Community-based Health Planning and Services)

For staff capacity, it was found that 48 midwives and 13 other cadre of health staff were recorded in the 26 facilities between 2015 and 2017; the 48 midwives served a total WIFA population of 157, 811, representing midwife: WIFA population ratio of 1: 3, 288 over the period. As shown in Table 2, the number of midwives per health facility ranged from none to highest of 12.

### Trend of maternal, newborn and child health (MNCH) services utilization

**Fig. 1:**
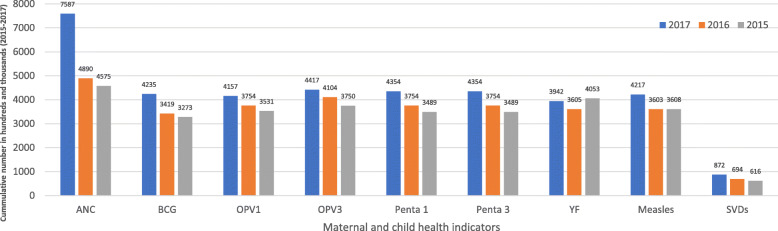
Aggregated yearly trend in maternal and child health services utilization (2015–2017). GHS/DHIMS II (Ho West District, 2015 – 2017); ANC (Antenatal Care); SVDs (Spontaneous Vaginal Deliveries); OPV1 (Oral Polio Vaccine 1); OPV3 (Oral Polio Vaccine 3); Penta1 (Pentavalent 1); Penta 3 (Pentavalent 3); YF (Yellow Fever)

Data from the DHIMSII show that a cumulative number of 2162 facility-based SVDs were recorded by 21 facilities between 2015 and 2017. Five (5) of the facilities did not have a record on facility-based SVD within the three-year period (2015–2017) in the DHIMSII; the highest cumulative facility-based SVDs over the 3 years was 445 in one clinic while the lowest was 3 in one CHPS compound, as shown in Table 2. Figure 1 further illustrates the yearly trend of MNCH services utilization from 2015 to 2017. Out of the 26 health facilities, 6 (23%) recorded SVDs even though did not have record of midwife at post which suggests possible unskilled deliveries. The WHO defines unskilled deliveries as births not attended to by doctors, nurses or midwives trained to provide life-saving maternal and newborn care during pregnancy, birth and the postnatal period. The definition includes deliveries by traditional birth attendants or by other auxiliary health workers trained to provide maternal and newborn care [2].

Fifteen (15) out of the 26 health facilities had concurring records of SVDs and midwives at post in the DHIMSII data. Out of this number, the highest cumulative number of SVDs per midwife within the period was 136 while the lowest was 7; one facility had three midwives at post but recorded no SVDs from 2015 to 2017 (see Table 2). Average SVDs per health facility appeared to be highest among urban facilities from 2015 to 2017 (see Table 4). Cumulative number of ANC visits recorded in the DHIMSII from 2015 to 2017 in the 26 facilities was 17,052, ranging from 0 to 3539 per health facility. Many more ANC visits were recorded in rural and public health facilities. Health centres recorded 9381 (55%) cumulative ANC visits from 2015 to 2017 followed by clinics 4282 (25%) and CHPS compounds 3389 (20%) (see Table 4).

**Table 4:** Average maternal and child health service utilization per health facility: disaggregated by facility location and reference year

	2015		2016		2017	
	Rural	Urban	Rural	Urban	Rural	Urban
**Child immunizations**	**Mean (95% CI)**	**Mean (95% CI)**	**Mean (95% CI)**	**Mean (95% CI)**	**Mean (95% CI)**	**Mean (95% CI)**
BCG	120 (82 158)	376 (58 694)*	136 (99 174)	210 (−6,101 029)*	149 (103 196)	403 (212 594)*
OPV1	126 (94 159)	440 (−6,271 507)*	142 (109 175)	313 (141 484)*	144 (107 181)	425 (−2,101 060)*
OPV3	136 (104 168)	445 (− 6,671 556)*	153 (117 189)	371 (333 409)*	151 (114 189)	471 (−3,111 252)*
Penta 1	125 (93 158)	429 (−4,991 357)*	142 (109 175)	313 (141 484)*	151 (111 191)	438 (− 3,571 232)*
Penta 3	125 (93 158)	429 (−4,991 357)*	142 (109 175)	313 (141 484)*	151 (111 191)	438 (−3,571 232)*
Yellow Fever	143 (103 183)	528 (− 1264 2320)*	134 (102 166)	334 (137 530)*	134 (103 166)	427 (− 114 967)*
Measles	126 (92 159)	485 (− 7,541 723)*	135 (103 166)	321 (289 352)*	146 (109 183)	427 (−114 967)*
WIFA population	1865 (−2,864 017)	3305 (−12,794 19,404)	1912 (− 2923 4117)	3389 (− 12,920 9697)*	1955 (− 2,964 207)	3423 (−12,645 19,490)
ANC attendance	115 (43 187)	907 (− 1,921 3734)*	138 (78 198)	791 (− 3002 4583)*	220 (161 280)	1148 (− 1050 3346)*
SVDs	21 (4 38)	90 (− 418 598)*	23 (9 36)	100 (− 644 843)*	27 (16 39)	125 (− 288 537)*

Source: GHS/DHIMS II (Ho West District, 2015–2017)

Legend: *ANC* (Antenatal Care); *SVDs* (Spontaneous Vaginal Deliveries); *WIFA* (Women in Fertility Age); *OPV1* (Oral Polio Vaccine 1); *OPV3* (Oral Polio Vaccine 3); *Penta1* (Pentavalent 1); *Penta 3* (Pentavalent 3)

*p < 0.001 (Poisson regression) statistically significant

**Fig. 2:**
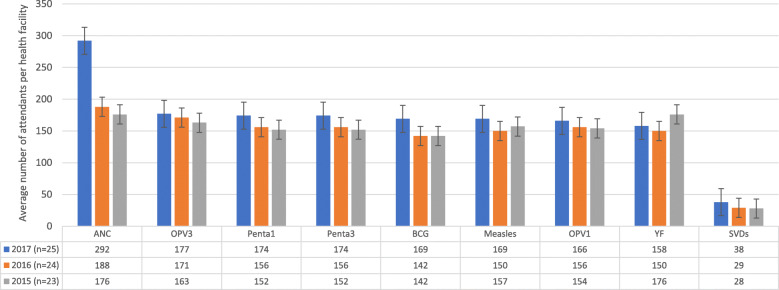
Average maternal and child health service utilization per health facility: disaggregated by reference year (2015–2017). GHS/DHIMS II (Ho West District, 2015 – 2017); ANC (Antenatal Care); SVDs (Spontaneous Vaginal Deliveries); WIFA (Women in Fertility Age); IRR (Incidence Rate Ratio); CI (Confidence Interval); OPV1 (Oral Polio Vaccine 1); OPV3 (Oral Polio Vaccine 3); Penta1 (Pentavalent 1); Penta 3 (Pentavalent 3); n (number of health facilities)

Even though the cumulative number of SVDs was 2162, the BCG immunizations record was 10,787 within the same period. Out of the 26 facilities, four (15%) did not have record any of the childhood immunizations (see Table 3). Average child immunizations per health facility increased marginally over the years (see Fig. 2) with consistently lower BCG service utilization and higher OPV3 utilization (see Table 3). Urban health facilities recorded relatively higher child immunizations than rural health facilities between 2015 and 2017 (see Table 4).

### Association between facility-based SVDs and newborn immunization records

**Fig. 3:**
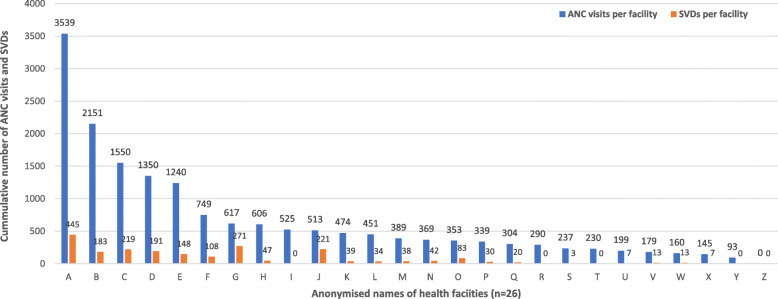
Association between number of ANC visits per health facility and SVDs record in the pertinent health facility (2015–2017). GHS/DHIMS II (Ho West District, 2015 – 2017); SVDs (Spontaneous Vaginal Deliveries); BCG (Bacillus-Calmette Guerin); ANC (Antenatal Care). GHS/DHIMS II (Ho West District, 2015 – 2017; SVDs (Spontaneous Vaginal Deliveries); BCG (Bacillus-Calmette Guerin); ANC (Antenatal Care)

**Fig. 4:**
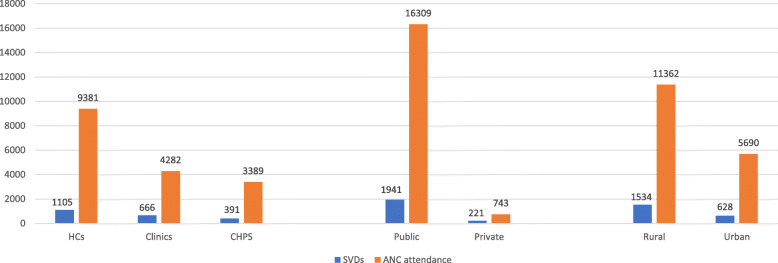
Association between number of ANC visits and SVDs: disaggregated by facility ownership, level and location (2015–2017). GHS/DHIMS II (Ho West District, 2015 – 2017); SVDs (Spontaneous Vaginal Deliveries); BCG (Bacillus-Calmette Guerin)

Figure 3 shows the association between number of ANC visits and SVDs per health facility from 2015 to 2017. The DHIMSII records revealed positive association between number of ANC visits recorded in a health facility and the number of SVDs recorded in the pertinent health facility. Thus, facilities which recorded higher ANC visits from 2015 to 2017 correspondingly recorded higher SVDs and vice versa. In terms of level of care, a positive correlation was also observed between number of ANC visits in a health facility and the number of SVDs recorded in the particular health facility. Publicly owned health centres located in rural areas also recorded higher ANC visits with corresponding high SVDs relative to their comparators, as illustrated in Fig. 4.

**Fig. 5:**
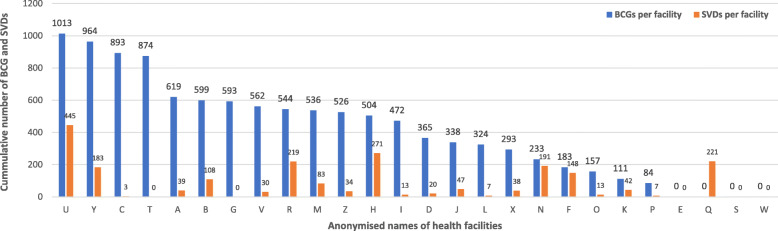
Correlation between cumulative SVDs records and child immunizations (2015–2017). GHS/DHIMS II (Ho West District, 2015 – 2017); SVDs (Spontaneous Vaginal Deliveries); BCG (Bacillus-Calmette Guerin)

**Fig. 6:**
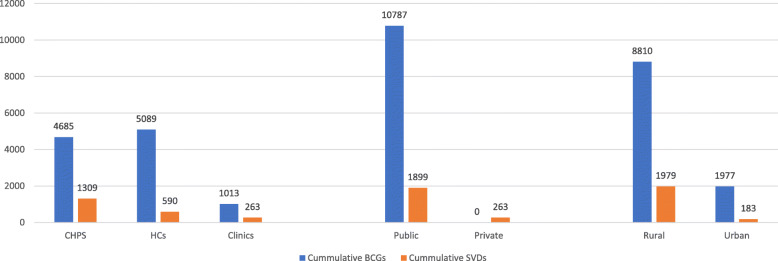
Correlation between cumulative SVDs records and child immunizations: disaggregated by facility ownership, level and location (2015–2017). GHS/DHIMS II (Ho West District, 2015 – 2017); SVDs (Spontaneous Vaginal Deliveries); BCG (Bacillus-Calmette Guerin)

Total of 2162 SVDs were recorded in the 26 health facilities from 2015 to 2017 compared to BCG record of 10,787 within the same time period. A shown in Fig. 5, number of facility-based SVDs recorded in a pertinent health facility did not match with the number of BCGs given at birth; one facility recorded 221 SVDs but had no record of BCG immunizations while another facility recorded 874 BCG immunizations without a record of SVD. Figure 6 shows that cumulatively, higher BCGs and SVDs were recorded in rural facilities relative to urban facilities; public facilities equally recorded higher BCGs and SVDs than private facilities; similarly, higher BCG immunizations were recorded in health centres than CHPS compounds and clinics even though there was no correlation with the cumulative number of SVDs recorded.

### Correlates of maternal, newborn and child health (MNCH) services utilization

Poisson regression model specification in Table 5 was used to predict the determinants of MNCH services utilization from the DHIMSII data. Output of the regression showed that a unit increase in the number of midwives in a health facility correspondingly increases the number of ANC visits (IRR = 1.30, [95% CI:1.29, 1.32]; *p* = 0.0001), and facility-based SVDs (IRR = 1.30 [95% CI:1.25, 1.35]; *p* = 0.0001) in the pertinent health facility, holding other covariates constant. Additionally, a positive correlation was found between high records of ANC visits in a health facility and high number of facility-based SVDs (IRR = 1.00 [95% CI:0.99, 1.00]; *p* = 0.0001) besides the number of midwives (IRR = 1.14 [95% CI:1.09, 1.20]; *p* = 0.0001) and other cadre of frontline health staff (IRR = 1.06 [95% CI:1.05, 1.07]; *p* = 0.0001) (see Table 6).

**Table 5:** Model specification for Poisson regression analyses: disaggregated by reference year (2015–2017)

		Summary statistics					
Variables	Variable definition	2015		2016		2017	
		**Obs.**	**Mean (SD)**	**Obs.**	**Mean (SD)**	**Obs.**	**Mean (SD)**
**Outcome variables (maternal)**							
ANC attendance	Number of women attending ANC	23	27 (43)	24	29 (41)	25	35 (38)
SVDs	Number of supervised deliveries for SVDs	23	142 (108)	24	142 (86)	25	169 (125)
**Outcome variables (child)**							
OPV1	Number of children receiving OPV1 vaccine	23	154 (116)	24	156 (86)	25	166 (113)
OPV3	Number of children receiving OPV3 vaccine	23	163 (114)	24	171 (99)	25	177 (123)
Penta1	Number of children receiving Penta 1 vaccine	23	152 (113)	24	156 (86)	25	174 (120)
Penta3	Number of children receiving Penta 3 vaccine	23	152 (113)	24	156 (86)	25	174 (120)
Yellow fever (YF)	Number of children receiving YF vaccine	23	176 (146)	24	150 (89)	25	158 (108)
Measles	Number of children receiving measles vaccine	23	157 (146)	24	150 (86)	25	169 (113)
**Covariates**							
Public^a^	1 of public health facility	26	0.92 (0.27)	26	0.92 (0.27)	26	0.92 (0.27)
Private	0 if private health facility (Reference)	26	0.08 (0.27)	26	0.08 (0.27)	26	0.08 (0.27)
Rural	1 if rural health facility	26	0.92 (0.27)	26	0.92 (0.27)	26	0.92 (0.27)
Urban	0 if urban health facility (Reference)	26	0.08 (0.27)	26	0.08 (0.27)	26	0.08 (0.27)
Midwives per clinic	Number of midwives per clinic	26	0.23 (0.43)	26	0.73 (0.87)	26	0.88 (1.37)
Other staff	Number of other health staff per clinic	25	3 (2)	25	3 (3)	25	4 (4)
WIFA population	Number of catchments WIFA population	26	1976 (4915)	26	2025 (5038)	26	2068 (5142)

Source: GHS/DHIMS II (Ho West District, 2015–2017)

Legend: *ANC* (Antenatal Care); *SVDs* (Spontaneous Vaginal Deliveries); *WIFA* (Women in Fertility Age); *IRR* (Incidence Rate Ratio); *CI* (Confidence Interval); *OPV1* (Oral Polio Vaccine 1); *OPV3* (Oral Polio Vaccine 3); *Penta1* (Pentavalent 1); *Penta 3* (Pentavalent 3); *SD* (Standard Deviation)

^a^Variable dropped from Poisson regression models for multicollinearity

**Table 6:** Determinants of maternal healthcare services utilization based on cumulative three-year reference data (2015–2017)

**Midwives per clinic and ANC attendance (Poisson Regression Model 1)**	**Dependent Variable: ANC (2015–2017)**		
**Independent Variables**	**IRR**	**(95% CI)**	***p*-value**
Midwives per clinic	1.30	1.29 1.32	0.000
WIFA population	0.99	0.99 1.00	0.000
Other staff per clinic	1.05	1.04 1.05	0.000
Rural	1.24	1.15 1.34	0.000
Urban	1.0	1.0	1.0
Log pseudolikelihood	− 577.75		
LR chi2(4)	1663.40		
Prob > chi2	0.0000		
Pseudo R2	0.5901		
Obs.	23		
**Midwives per clinic and SVDs (Poisson Regression Model 2)**	**Dependent Variable: SVDs (2015–2017)**		
**Independent Variables**	**IRR**	**(95% CI)**	***p*-value**
Midwives per clinic	1.30	1.25 1.35	0.000
WIFA population	0.99	0.99 1.00	0.000
Other staff per clinic	1.08	1.07 1.09	0.000
Rural	0.35	0.19 0.65	0.001
Urban	1.0	1.0	1.0
Log pseudolikelihood	−577.75132		
LR chi2(4)	1663.40		
Prob > chi2	0.0000		
Pseudo R2	0.5901		
Obs.	23		
**ANC attendance and SVDs (Poisson Regression Model 3)**	**Dependent Variable: SVDs (2015–2017)**		
**Independent Variables**	**IRR**	**(95% CI)**	***p*-value**
ANC attendance	1.00	0.99 1.00	0.000
Midwives per clinic	1.14	1.09 1.20	0.000
WIFA population	0.99	0.99 1.00	0.000
Other staff per clinic	1.06	1.05 1.07	0.000
Rural	0.90	0.46 1.77	0.758
Urban	1.0	1.0	1.0
Log pseudolikelihood	−530.23193		
LR chi2(4)	1758.44		
Prob > chi2	0.0000		
Pseudo R2	0.6238		
Obs.	23		

Source: GHS/DHIMS II (Ho West District, 2015–2017)

Legend: ANC (Antenatal Care); SVDs (Spontaneous Vaginal Deliveries); WIFA (Women in Fertility Age); IRR (Incidence Rate Ratio); CI (Confidence Interval)

*p < 0.001 (Poisson regression) statistically significant

A positive correlation was also observed between the number of midwives per health facility and the number of childhood immunizations: BCG, OPV1, OPV3, Penta1, Penta3, YF and Measles (*p* < 0.0001); high number of midwives in a health facility also had a positive correlation with the number of records on child immunizations (*p* < 0.0001). Similarly, there was a positive association between rural location of a health facility and utilization of childhood immunizations relative to urban facilities (*p* < 0.0001), holding other covariates constant (see Table 7).

**Table 7:** Determinants of child health service utilization based on cumulative three-year reference data (2015–2017)

	Dependent Variables (child immunizations)						
Independent Variables	BCG (Univariate I)	OPV1 (Univariate II)	OPV3 (Univariate III)	Penta1 (Univariate IV)	Penta3 (Univariate V)	YF (Univariate VI)	Measles (Univariate VII)
	**IRR (95% CI)**	**IRR (95% CI)**	**IRR (95% CI)**	**IRR (95% CI)**	**IRR (95% CI)**	**IRR (95% CI)**	**IRR (95% CI)**
Midwives per clinic	1.32 (1.29 1.34)*	1.25 (1.22 1.27)*	1.25 (1.23 1.27)*	1.25 (1.23 0.27)*	1.25 (1.23 1.27)*	1.25 (1.22 1.27)*	1.25 (1.23 1.27)*
WIFA population	1.00 (1.00 1.00)*	1.00 (1.00 1.00)*	1.00 (1.00 1.00)*	1.00 (1.00 1.00)*	1.00 (1.00 1.000)*	1.00 (1.00 1.000)*	1.00 (1.00 1.00)*
Other staff per clinic	0.96 (0.96 0.97)*	0.97 (0.97 0.98)*	0.98 (0.98 0.98)*	0.97 (0.97 0.98)*	0.97 (0.97 0.98)*	0.97 (0.97 0.98)*	0.97 (0.97 0.98)*
Rural	68.56 (53.22 88.32)*	23.41 (18.30 29.96)*	22.21 (17.48 28.22)*	25.06 (19.62 32.01)*	25.06 (19.62 32.01)*	22.52 (17.60 28.82)*	24.23 (18.91 31.05)*
Urban	1.0	1.0	1.0	1.0	1.0	1.0	1.0
Log pseudolikelihood	− 564.57	− 515.93	− 616.18	− 517.88	− 517.88	− 510.56	− 496.04
LR chi2(4)	3310.25	3247.38	3014.44	3292.27	3292.27	3767.78	3548.59
Prob > chi2	0.0105	0.0000	0.0000	0.0000	0.0000	0.0000	0.0000
Pseudo R2	0.7457	0.7589	0.7098	0.7607	0.7607	0.7868	0.7815
Obs.	23	23	23	23	23	23	23
ANC attendance	1.00 (1.00 1.00)*	1.00 (1.00 1.00)*	1.00 (1.00 1.00)*	1.00 (1.00 1.00)*	1.00 (1.00 1.00)*	1.00 (1.00 1.00)*	1.00 (1.00 1.00)*
Midwives per clinic	1.24 (1.21 1.27)*	1.19 (1.17 1.22*	1.16 (1.14 1.19)*	1.19 (1.17 1.22)*	1.19 (1.17 1.22)*	1.19 (1.17 1.22)*	1.00 (1.00 1.00)*
WIFA population	1.00 (1.00 1.00)*	1.00 (1.00 1.00)*	1.00 (1.00 1.00)*	1.00 (1.00 1.00)*	1.00 (1.00 1.00)*	1.00 (1.00 1.00)*	1.20 (1.17 1.23)*
Other staff per clinic	0.96 (0.96 0.96)*	0.97 (0.97 0.98)*	0.98 (0.97 0.98)*	0.97 (0.97 0.97)*	0.97 (0.97 0.97)*	0.97 (0.97 0.98)*	1.00 (1.00 1.00)*
Rural	90.18 (69.42 7.16)*	28.64 (22.20 36.96)*	31.33 (24.44 0.16)*	30.87 (23.97 39.76)*	30.87 (23.97 39.76)*	27.74 (21.49 35.79)*	0.97 (0.97 0.98)*
Urban	1.0	1.0	1.0	1.0	1.0	1.0	1.0
Log pseudolikelihood	− 526.61	− 495.29	− 553.65	−495.41	− 495.41	− 488.83	− 477.05
LR chi2(4)	3386.18	3288.67	3139.51	3337.21	3337.21	3811.23	3586.56
Prob > chi2	0.0000	0.0000	0.0000	0.0000	0.0000	0.0000	0.0000
Pseudo R2	0.7628	0.7685	0.7393	0.7711	0.7711	0.7958	0.7899
Obs.	23	23	23	23	23	23	23
SVDs	1.00 (1.00 1.00)*	1.00 (1.00 1.00)*	1.00 (1.00 1.00)*	1.00 (1.00 1.00)*	1.00 (1.00 1.00)*	1.00 (1.00 1.00)*	1.00 (1.00 1.00)*
Midwives per clinic	1.29(1.26 1.32)*	1.22 (1.20 1.25)*	1.20 (1.18 1.23)*	1.22 (1.20 1.25)*	1.22 (1.20 1.25)*	1.22 (1.19 1.24)*	1.23 (1.20 1.26)*
WIFA population	1.00 (1.00 1.00)*	1.00 (1.00 1.00)*	1.00 (1.00 1.00)*	1.00 (1.00 1.00)*	1.00 (1.00 1.00)*	1.00 (1.00 1.00)*	1.00 (1.00 1.00)*
Other staff per clinic	0.96 (0.96 0.96)*	0.97 (0.97 0.98)*	0.98 (0.97 0.98)*	0.97 (0.97 0.97)*	0.97 (0.97 0.97)*	0.97 (0.97 0.98)*	0.97 (0.97 0.98)*
Rural	72.05 (55.80 3.03)*	24.39 (19.02 31.27)*	24.13 (18.94 30.74)*	26.14 (20.42 33.45)*	26.14 (20.42 3.45)*	23.74 (18.51 30.45)*	25.18 (19.61 32.32)*
Urban	1.0	1.0	1.0	1.0	1.0	1.0	1.0
Log pseudolikelihood	− 557.24	−510.62	− 592.31	− 512.20	−512.20	− 502.36	−491.71
LR chi2(4)	3324.93	3258.00	3062.20	3303.63	3303.63	3784.17	3557.25
Prob > chi2	0.0000	0.0000	0.0000	0.0000	0.0000	0.0000	0.0000
Pseudo R2	0.7490	0.7613	0.7211	0.7633	0.7633	0.7902	0.7834
Obs.	23	23	23	23	23	23	23

Source: GHS/DHIMS II (Ho West District, 2015–2017)

Legend: *ANC* (Antenatal Care); *SVDs* (Spontaneous Vaginal Deliveries); *WIFA* (Women in Fertility Age); *IRR* (Incidence Rate Ratio); *CI* (Confidence Interval)

*p < 0.001 (Poisson regression) statistically significant

## Discussion

Global efforts towards attainment of universal health coverage has been largely progressive, albeit the pace in low- and middle-income countries (LMICs) is comparatively slow [3]. Studies on continuum of care (CoC) for MNCH services abound in Ghana [7–12] but there are no known ecological studies on the topic area using the DHIMSII data set from a predominantly rural district in the Volta Region of Ghana. The existing gap in the literature thus makes this study relevant and timely.

Results from this study have demonstrated that MNCH service utilization, are significantly associated with rural-urban differentials and distribution of human and material health resources as alluded to in previous studies on Ghana [13–16] and other countries [16, 17]. Density of midwives and other frontline health staff significantly correlated with number of ANC visits recorded in the study health facilities over the three-year period, contrary to findings by similar studies on Ghana [9, 18, 19] and other countries [20, 21], which reported that the number of frontline health workers per se did not enhance utilization of maternal and child health services. These studies argue that poor attitudes of staff and other health system challenges remain important constraints to utilization of maternal, newborn and child health (MNCH) services in Ghana [16, 22–30].

Even though poor attitude of health staff towards their clients is widely documented as an important disincentive to many healthcare clients to utilize safer healthcare services, rural communities often do not have alternatives to the only available healthcare providers. Consequently, higher health services utilization in rural locations is not always a function of improved healthcare delivery system but a reflection of lack of choice for better alternatives. Indeed, other barriers to MNCH service utilization not explored in this study are acknowledged in terms of long travel times to health facilities [24, 26, 29], socio-cultural beliefs [16, 23, 30, 31] and financial inaccessibility [32–37].

This study therefore recognizes these existing challenges while underscoring the continued critical role of health sector human resources towards improving universal access to MNCH services [25, 27, 28, 38–41]. As demonstrated in this study, facility-based SVDs over the three-year period correlated positively with number of midwives in the pertinent health facility and the cumulative number of ANC visits recorded in the particular health facility. Sheff et al. [42] arrived at similar conclusions in their study on maternal and child health services utilization in the Volta Region.

The evidence demonstrated in this paper presents an empirical basis for Ghana to invest more in health sector human resources particularly as part of the strategic plan to achieve universal access to basic healthcare services. This long-term crucial investment, alongside ongoing interventions, could prove useful towards achieving the SDG 3 targets in Ghana and other resource constrained countries in Africa [3]. Although poor attitude of health professionals and the negative effect on universal access to healthcare remain a barrier to optimal health service utilization [22, 25, 27, 31], the available evidence suggests frontline health personnel remain an indispensable piece of the puzzle to overcoming this challenge in health systems. Indeed, the WHO tenets on universal health coverage emphasizes the vital role of frontline healthcare professionals in transforming health systems across the globe [38].

Another revelation from the analyzed data was the fact that consistently, the cumulative numbers of facility-based SVDs, ANC visits and child immunizations increased marginally from 2015 to 2017. The trend largely reflects the national picture in Ghana which portrays a yearly marginal increase in the utilization of MNCH services [41].

The positive association observed between high ANC visits and facility-based SVDs in the DHIMSII data also corroborates conclusions in previous studies on Ghana. Systematic reviews [7, 10] and empirical studies [11–13, 17, 22] on Ghana observed that mothers who recorded at least four (4) ANC visits were more likely to experience facility-based SVD and remain in the CoC for postnatal care including child health services than mothers who recorded less ANC visits.

Finally, it is imperative to state that this study design differs significantly from these previous studies and does not allow for direct comparison of the outcomes. Also, conclusions on retention and dropout from the CoC cannot be made in this study because, the data set was not on individual registrants or attendants. Instead, the data entailed aggregated cumulative figures from the 26 primary healthcare facilities in the study district. In light of this acknowledged limitation, individual level conclusions will amount to an ecological fallacy since generalizations cannot be made on individuals from the pool of data reported in the DHIMSII. Nonetheless, the cumulative data from the various health facilities gives a global picture of trends and associated factors of MNCH services utilization. This wholistic view will help to inform district level health policy decisions and trigger follow-up empirical studies to understand specific facility level details on the topic.

## Conclusion

Coverage for maternal and child health services in the study district marginally increased over the three-year period, albeit evidence of 23% of the study facilities recording unskilled deliveries was discovered. The aggregate DHIMSII data found compelling evidence of a positive association between high records of ANC visits in the study facilities and the cumulative records of facility-based SVDs; likewise, higher records of ANC visits and facility-based SVDs correlated positively with the cumulative number of child immunizations. Additionally, number of frontline health staff (especially midwives) in a health facility and geographic location of the pertinent facility had positive associations with MNCH services utilization. Although these findings are largely informed by ecological population-based aggregate data, the evidence remain compelling and should stimulate actions for district level policy dialogues and further epidemiological enquiries through mixed-methods research designs to unearth facility-level peculiarities on the determinants of MNCH services utilization in the study region and possibly replicated to other parts of the country.

### Implications for health policy

In light of the above findings, the following policy recommendations are proposed:

1. DHIMSII data remains a vital data source that should be leveraged to informed evidence-based policy decisions backed by relevant empirical studies. Annual regional performance reviews should therefore compel regions and districts to report on scientific outputs from the DHIMSII as part of their performance assessment criteria.
2. Unskilled deliveries remain a challenge in many deprived districts in Ghana as demonstrated in this study, there is therefore the need for enhanced efficiencies in the allocation and distribution of skilled personnel to improve the situation. Targeted staff motivation schemes will attract and retain qualified health staff to deprived communities in the country.
3. Therefore is the need for accelerated targeted support system for pregnant mothers and their partners to help in enrollment and retention of mothers in the CoC for MNCH services.

### Limitations of the study

This study relied mainly on ecological population-based data from DHIMSII without the benefit of complementary primary data to unearth clients’ and healthcare providers’ personal experiences in respect of service quality gaps and the consequent effect on utilization of MNCH services. Conclusions from this study are therefore not informed by individual personal health information. The figures on MNCH service indicators are cumulative aggregates reported over a three-year period in all the 26 facilities in the study district. Also, since the DHIMSII data has limitations of possible entry errors and misreporting that are often difficult to detect, perhaps some of the figures might have been over or under reported.

Finally, the results of the DHIMSII might have been skewed by the case management trends of these lower level facilities. Nonetheless, the three-year data period (2015–2017) gives reasonable global picture of the MNCH services utilization trends which is capable of informing further similar investigations in other districts and regions. Future research could therefore consider triangulating primary and secondary data collection approaches for a better appreciation of the depth of this explorative endeavour.

## Supplementary information

**Additional file 1.**

## Data Availability

All relevant data are within the manuscript and its Supporting Information files.

## Abbreviations

ANC: Antenatal care
BCG: Bacillus calmette guerin
CoC: Continuum of care
CHPS: Community-based health planning and services
CHO: Community health officer
DHIMS II: District health information management system II
DPT: Diphtheria pertussis tetanus
GHS: Ghana health service
HWD: Ho west district
IRR: Incidence rate ratio
MMR: Maternal mortality ratio
MNCH: Maternal, newborn and child health
MoH: Ministry of health
OLS: Ordinary least squares
OPV1: Oral polio vaccine 1
OPV3: Oral polio vaccine 3
Penta1: Pentavalent 1
Penta3: Pentavalent 3
PHC: Population and housing census
SDG: Sustainable development goals
SVDs: Spontaneous vaginal deliveries
VIF: Variance inflation factor
WHO: World health organization
WIFA: Women in fertile age
YF: Yellow fever
